# Comparing the Ability of Secretory Signal Peptides for Heterologous Expression of Anti-Lipopolysaccharide Factor 3 in *Chlamydomonas reinhardtii*

**DOI:** 10.3390/md21060346

**Published:** 2023-06-04

**Authors:** Huilin Zhuang, Yaohui Ou, Ruoyu Chen, Danqiong Huang, Chaogang Wang

**Affiliations:** 1 Guangdong Technology Research Center for Marine Algal Bioengineering, College of Life Sciences and Oceanography, Shenzhen University, Shenzhen 518060, China; 2 Shenzhen Engineering Laboratory for Marine Algal Biological Development and Application, College of Life Sciences and Oceanography, Shenzhen University, Shenzhen 518060, China; 3 Laboratory of Marine Bioresource & Eco-Environmental Science, College of Life Sciences and Oceanography, Shenzhen University, Shenzhen 518060, China; 4 Guangdong Provincial Key Laboratory for Plant Epigenetics, College of Life Sciences and Oceanography, Shenzhen University, Shenzhen 518060, China

**Keywords:** antimicrobial peptide, *Chlamydomonas reinhardtii*, anti-lipopolysaccharide factor 3, secretory expression, arylsulfatase 1 signal peptide, carbonic anhydrase 1 signal peptide

## Abstract

Anti-lipopolysaccharide factor 3 (ALF*Pm*3) possesses a wide antimicrobial spectrum and high antibacterial and viral activities for broad application prospects in the aquaculture industry. However, the application of ALF*Pm*3 is limited by its low production in nature, as well as its low activity when expressed in *Escherichia coli* and yeast. Although it has been proven that its secretory expression can be used to produce antimicrobial peptides with strong antimicrobial activity, there is no study on the high-efficiency secretory expression of ALF*Pm*3 in *Chlamydomonas reinhardtii*. In this study, signal peptides ARS1 and CAH1 were fused with ALF*Pm*3 and inserted into the pESVH vector to construct pH-aALF and pH-cALF plasmids, respectively, that were transformed to *C. reinhardtii* JUV using the glass bead method. Subsequently, through antibiotic screening, DNA-PCR, and RT-PCR, transformants expressing ALF*Pm*3 were confirmed and named T-JaA and T-JcA, respectively. The peptide ALF*Pm*3 could be detected in algal cells and culture medium by immunoblot, meaning that ALF*Pm*3 was successfully expressed in *C. reinhardtii* and secreted into the extracellular environment. Moreover, ALF*Pm*3 extracts from the culture media of T-JaA and T-JcA showed significant inhibitory effects on the growth of *V. harveyi*, *V. alginolyticus*, *V. anguillarum*, and *V. parahaemolyticus* within 24 h. Interestingly, the inhibitory rate of c-ALF*Pm*3 from T-JcA against four Vibrio was 2.77 to 6.23 times greater than that of a-ALF*Pm*3 from T-JaA, indicating that the CAH1 signal peptide was more helpful in enhancing the secreted expression of the ALF*Pm*3 peptide. Our results provided a new strategy for the secretory production of ALF*Pm*3 with high antibacterial activity in *C. reinhardtii*, which could improve the application potentiality of ALF*Pm*3 in the aquaculture industry.

## 1. Introduction

The abuse of antibiotics leads to the frequent occurrence of drug-resistant microorganisms and brings irreversible losses in the aquaculture industry [1,2]. The prohibition of the addition of antibiotics in breeding feed is a consensus. Antimicrobial peptides (AMPs) originally found in insects [3] have become the perfect substitutes for antibiotics due to their broad antimicrobial spectrum, strong antibacterial activity, and low susceptibility to drug resistance [4,5,6]. As one cluster of the known AMPs, anti-lipopolysaccharide factors (ALF) firstly found in *Limulus polyphemus* displayed high antibacterial activity [7]. In recent years, it was reported that the anti-lipopolysaccharide factor 3 (ALF*Pm*3) from *Penaeus monodon* with a lipopolysaccharide binding domain (LBD) had strong antibacterial activity against common aquaculture pathogenic bacteria, such as *Vibrio harveyi*, *Vibrio parahaemolyticus*, and *Staphylococcus aureus*, at a very low concentration (0.77 µM), showing its potential for application in the aquaculture industry [8,9,10]. ALF*Pm*3 is commonly produced via genetic engineering due to its low yield from natural sources. However, ALF*Pm*3 is difficult to express in *Escherichia coli* because of its high antimicrobial activity against *E. coli* [8,11]. Although ALF*Pm*3 could be expressed in *P. pastoris*, its antibacterial activity is affected by excessive glycosylation in *P. pastoris* [12,13]. With lots of advantages for industry, such as short growth cycle, posttranslational modification, low and cheap nutritional requirements, and efficient large-scale culture techniques [14,15,16], microalgae have been received widespread attention for their potential to be a factory to produce valuable components. Currently, studies on microalgae are mainly focused on *Chlamydomonas reinhardtii*, *Chlorella vulgaris*, *Phaeodactylum tricornutum*, and so on. However, in *Chlorella* and *P. tricornutum*, the expression of exogenous genes is unstable and the transformation efficiency is low [17,18]. Differently, as a model species, genetic manipulation toll kits were well developed in *C. reinhardtii* [19]. In addition, *C. reinhardtii* has the advantages of high photosynthesis efficiency, no endotoxin, and reliable industrial cultivation system [20]. It was reported that several medicinal proteins, such as the RBD vaccine [21,22], IFN-α [23], and human interleukin 2 [24], were successfully expressed in *C. reinhardtii,* indicating that *C. reinhardtii* is an ideal platform for the production of pharmaceutical proteins.

Until now, the genetic transformation of foreign genes in the nuclei, chloroplasts, and mitochondria of *C. reinhardtii* has been reported. However, target products were mainly accumulated within cells. In general, the extracellular secretory expression system can help secrete proteins into culture media, providing convenience for protein isolation, purification, and industrial application [25,26]. Moreover, glycosylation modification during the secretion process also facilitates the stability of the protein structure [27,28]. It is a pity that only a few studies on the secretion of proteins have been conducted on *C. reinhardtii*. Nowadays, secretory expression is mainly achieved through signal peptides (SPs), which are located at the N-terminal of proteins and carry protein secretory information to mediate the secretion process. Different SPs perform differently in the secretion and production of recombinant proteins. For example, a 10-fold difference on the secretion of the mCherry fluorescent protein was observed among more than 2000 signal peptides [29]. Even more significantly, a 100-fold difference in the protein secretion in CHO cells was found among 17 signal peptides [30]. Therefore, finding effective SPs is important for the development of the secretory expression system in *C. reinhardtii*.

Previously, it was reported that over 95% of the xylanase 1 produced in *C. reinhardtii* was successfully secreted into the culture medium by inserting arylsulfatase 1 (ARS1) into the coding region of *xyn1* [27]. Additionally, the secretion of luciferase in *C. reinhardtii* was increased by nearly 84% when using carbonic anhydrase 1 (CAH1) [31]. Therefore, ARS1 and CAH1 signal peptides have a great potential in mediating the secretion of ALF*Pm*3 in *C. reinhardtii*.

In this study, ARS1 and CAH1 were fused with ALF*Pm*3 to construct plasmids pH-aALF and pH-cALF, respectively, which were then transferred into the nuclear genome of *C. reinhardtii* using the glass bead method. Transformants were identified through DNA-PCR, RT-PCR, and immunoblot analysis. Furthermore, their ability to inhibit aquaculture pathogenic bacteria was also evaluated. Results showed that ALF*Pm*3 could be secreted into medium with the help of the ARS1 signal peptide and the CAH1 signal peptide. The protein extracts from the medium showed high antibacterial activity towards aquaculture pathogenic bacteria such as *Vibrio harveyi*, *Vibrio alginolyticus*, *Vibrio anguillarum*, and *Vibrio parahaemolyticus*. This study provides a new idea for the highly efficient expression and secretion of AMPs in *C. reinhardtii.*

## 2. Results

### 2.1. Design of ALFPm3 Expression Cassette

**Figure 1:**
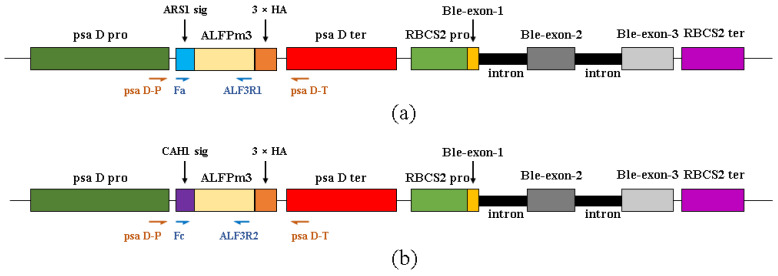
The diagram of the “ARS1-ALF*Pm*3” and “CAH1-ALF*Pm*3” expression cassettes. (**a**) “ARS1-ALF*Pm*3” expression cassette in plasmid pH-aALF. (**b**) “CAH1-ALF*Pm*3” expression cassette in plasmid pH-cALF. PsaD pro: the psaD promoter, PsaD ter: the psaD terminator, RBCS2 pro: the RBCS2 promoter, RBCS2 ter: the RBCS2 terminator, Ble-exon-1,2,3: the three exons of *ble* gene, intron: the intron of *RBCS2* gene, ARS1 sig: ARS1 signal peptide, CAH1 sig: CAH1 signal peptide, 3 × HA: 3 × HA tag for protein identification. PsaD-P and psaD-T were primers used for genomic PCR. Fa and ALF3R1 were primers used for RT-PCR of T-JaA. Fc and ALF3R2 were primers used for RT-PCR of T-JcA.

Based on the codon preference of the *C. reinhardtii* nuclear genome, the ALF*Pm*3 gene from *P. monodon* (Genbank number JQ256520) was optimized and its GC content was increased from 56% to 68%. The signal peptides ARS1 (21aa: MGALAVFAVACLAAVASVAHA) and CAH1 (23aa: MARTGALLLVALALAGCAQACIY) were fused with ALF*Pm*3 at the N-terminal, respectively. The 3 × HA tag was connected to the C-terminal of ALF*Pm*3 for subsequent protein identification. The psaD promoter and psaD terminator with high efficiency in expressing heterologous genes in *C. reinhardtii* [32,33] were used to express target gene. The expression cassette of the *ble* given transformants with the bleomycin/zeocin-resistance was used as the selection maker [19,34]. To screen positive transformants at molecular level, the psaD-P and psaD-T primer set, located in the psaD promoter and psaD terminator region, was used. The Fa and ALF3R1 and the Fc and ALF3R2 primer sets, located in the signal peptide region and ALF*Pm*3 coding region, were used for RT-PCR to confirm the expression of ALF*Pm*3 in transformants (Figure 1).

### 2.2. Screening of Transgenic C. reinhardtii

The transformation of *C. reinhardtii* JUV was carried out using the glass bead method. After cultivation for three to four weeks, green colonies were visible and were transferred to the new TAP agar medium containing 100 µg/mL ampicillin and 10 µg/mL zeocin. Candidates of algae transformed with plasmid pH-aALF were named T-JaA, and those with plasmid pH-cALF were named T-JcA. It was found that the transformation frequency was 2.5 × 10^−5^ in T-JaA and 3.7 × 10^−5^ in T-JcA.

**Figure 2:**
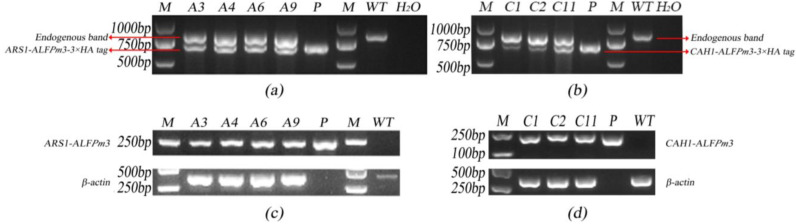
Genomic PCR analysis and RT-PCR analysis of transgenic algae. (**a**) Genomic PCR analysis of T-JaA. (**b**) Genomic PCR analysis of T-JcA. (**c**) RT PCR analysis of T-JaA. (**d**) RT PCR analysis of T-JcA. Endogenous band: Genomic PCR analysis of the endogenous gene of *C. reinhardtii*. ARS1-ALF*Pm*3-3 × HA tag: Genomic PCR analysis of *ARS1-ALFPm3-3 × HA tag* gene in T-JaA. CAH1-ALF*Pm*3-3 × HA tag: Genomic PCR analysis of *CAH1-ALFPm3-3 × HA tag* gene in T-JcA. ARS1-ALF*Pm*3: RT-PCR analysis of *ARS1-ALFPm3* gene in T-JaA. CAH1-ALF*Pm*3: RT-PCR analysis of *CAH1-ALFPm3* gene in T-JcA. β-actin: RT-PCR analysis of *β-actin* gene. M: DL 2000 DNA ladder marker. P: Positive control. WT: *C. reinhardti*i JUV. H_2_O: blank control. A3, A4, A6, A9: Candidates of T-JaA. C1, C2, C11: Candidates of T-JcA.

As a common endogenous promoter of microalgae, the native psaD promoter/terminator is responsible for initiating the transcription of photosystem I complex related proteins in *C. reinhardtii* [33]. Therefore, PCR with primers psaD-P and psaD-T could generate an 813 bp endogenous gene fragment in the genome of *C. reinhardtii* while generating additional 679 bp and 676 bp fragments in T-JaA and T-JcA, respectively. As expected, according to the genomic PCR results, positive transformants could detect target bands at both 679/676 bp and 813 bp, indicating that the target genes were successfully inserted into the genome of *C. reinhardtii* JUV (Figure 2a,b). 

The expression of ALF*Pm*3 in T-JaA and T-JcA was identified by RT-PCR using primer pairs Fa/ALF3R1 and Fc/ALF3R2, respectively. PCR fragments at 222 bp and 203 bp were expected in T-JaA and T-JcA, respectively. As expected, the results of RT-PCR showed that target fragments could be detected as the positive control (Figure 2c,d), indicating the successful expression of ALF*Pm*3 at the transcription level in *C. reinhardtii* JUV.

### 2.3. Analysis of Intracellular and Extracellular Expression of ALFPm3 in Transgenic 

#### *C. reinhardtii* 

**Figure 3:**
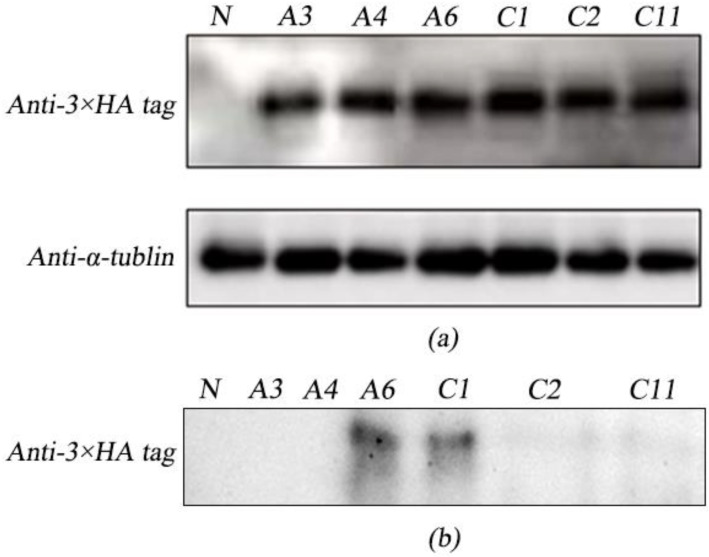
The immunoblot analysis of intracellular and secretory proteins extracted from genetically engineered algae T-JaA and T-JcA. (**a**) Immunoblot analysis of proteins from algal cells of T-JaA and T-JcA. (**b**) Immunoblot analysis of proteins from the culture medium of T-JaA and T-JcA. Anti-3 × HA tag: protein extract was incubated with mouse anti-3 × HA tag antibody for testing the existence of 3 × HA tag proteins. Anti-α-tublin: protein extract was incubated with mouse anti-α-tublin tag antibody for testing the existence of α-tublin. N: Negative control. A3, A4, A6: Candidates of T-JaA. C1, C2, C11: Candidates of T-JcA.

The existence of ARS1-ALF*Pm*3 and CAH1-ALF*Pm*3 fusion proteins in cells was evaluated by immunoblot. Results showed that the proteins ARS1-ALF*Pm*3 (a-ALF*Pm*3) and CAH1-ALF*Pm*3 (c-ALF*Pm*3) were detected in T-JaA (A3, A4, A6) and T-JcA (C1, C2, C11), respectively, which was evidenced by the detection of 3 × HA using anti-3 × HA tag antibody at the expected size of about 16 kD and no detection of that in in the wide type of *C. reinhardtii* JUV (WT) (Figure 3a). Meanwhile, the protein of β-actin was detected in all the wild-type and positive transformants using anti-α-tublin antibody (Figure 3a), indicating that ARS1-ALF*Pm*3 and CAH1-ALF*Pm*3 fusion proteins were successfully expressed in transgenic *C. reinhardtii* JUV.

Subsequently, the extracellular secretion of fusion proteins a-ALF*Pm*3 and c-ALF*Pm*3 was analyzed in culture medium. Results showed that only proteins extracted from the culture medium of T-JaA6 (A6) and T-JcA1 (C1) generated the hybrid signal at 16 kD using anti-3 × HA tag antibody (Figure 3b), indicating that the ALF*Pm*3 peptide was successfully secreted in vitro when fused with signal peptides ARS1 and CAH1. However, the target protein was not detected in the culture medium of A3, A4, C2, and C11, even though target proteins were found within cells (Figure 3a). A6 and C1 were used for subsequent bacteriostatic analysis.

### 2.4. Both a-ALFPm3 and c-ALFPm3 Showed High Antibacterial Activity

**Figure 4:**
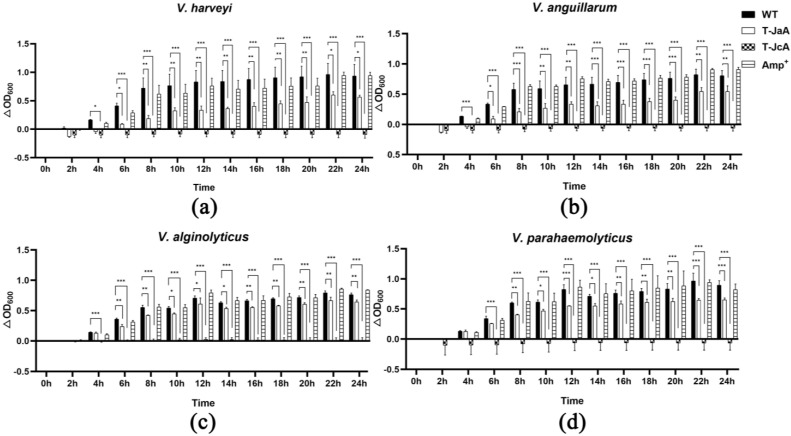
The effects of protein extracts from T-JaA6 and T-JcA1 on the growth of aquaculture pathogenic bacteria. The data were analyzed statistically. (**a**) *V. harveyi.* (**b**) *V. anguillarum.* (**c**) *V. alginolyticus.* (**d**) *V. parahaemolyticus.* WT: culture supernatant of *C. reinhardtii* JUV. T-JaA6: culture supernatant of T-JaA6. T-JcA1: culture supernatant of T-JcA1. Amp^+^: ampicillin (2 mg/mL).*, **, ***: statistically difference at the level of 0.05, 0.01, and 0.001, respectively.

For the antibacterial analysis, 100 mL of culture medium of T-JaA6 and T-JcA1 was collected and freeze dried for protein extraction. The concentration of protein extracts from T-JaA6 (containing a-ALF*Pm*3) was 0.98 µg/µL, and that from T-JcA1 (containing c-ALF*Pm*3) was 2.02 µg/µL. Compared with the wild type, protein extracts containing a-ALF*Pm*3 and c-ALF*Pm*3 had significant inhibitory effects on the growth of *V. harveyi*, *V. alginolyticus*, *V. anguillarum*, and *V. parahaemolyticus* within 24 h (Figure 4). After 24 h, except for *V. alginolyticus* with a bacteriostatic rate of 15.79%, the bacteriostatic rate of a-ALF*Pm*3 against aquaculture pathogenic bacteria exceeded 30%. The highest bacteriostatic rate, 39.67%, was found against *V. harveyi*. It is noted that c-ALF*Pm*3 performed much better than a-ALF*Pm*3, which had a bacteriostatic rate of over 98% against all four aquaculture pathogenic bacteria. Similarly, the highest bacteriostatic rate was found against *V. harveyi*, reaching 110.07%. The two types of ALF*Pm*3 had the strongest inhibitory effect on *V. harveyi* and the weakest inhibitory effect on *V. alginolyticus*. Although the concentration of ampicillin used in the study was as high as 2 mg/mL, its inhibitory efficiency was only −0.69%, −9.90%, −12.77%, and 8.48% against *V. harveyi*, *V. alginolyticus*, *V. anguillarum*, and *V. parahaemolyticus,* respectively, after 24 h. The antibacterial activity of a-ALF*Pm*3 and c-ALF*Pm*3 against *V. parahaemolyticus* was 3.88 and 12.67 times as that of ampicillin, demonstrating the strong antibacterial activity of a-ALF*Pm*3 and c-ALF*Pm*3. Indeed, the bacteriostatic effect of ALF*Pm*3 was also affected by the type of signal peptide, and after 24 h, the inhibitory rate of c-ALF*Pm*3 against *V. harveyi*, *V. alginolyticus*, *V. anguillarum*, and *V. parahaemolyticus* was 2.77, 6.23, 3.32, and 3.26 times that of a-ALF*Pm*3, indicating that the CAH1 signal peptide was more effective in secreting the ALF*Pm*3 peptide in *C. reinhardtii* than the ARS1 signal peptide.

## 3. Discussion

Prokaryotic expression systems such as *E. coli* were not generally used to express AMPs, since those AMPs with high antibacterial activity might kill *E.coli* [35,36,37]. Given the previous studies, *P. pastoris* successfully expressed and secreted some AMPs, such as rFcALF2 [38], rMnALF4 [39], Mytichitin-A [40], and rPaDef [41]. However, the secretory expression of rALF*Pm*3 in *P. pastoris* was only found in a small quantity outside the cell [42]. Additionally, the activity of recombinant proteins secreted by *P. pastoris* was affected, for example, compared with the natural proteins Mycithin-A [40], CecropinB2 [43], and Ch-Penaeidin [44] secreted by *P. pastoris*, which had no or low antibacterial activity. Although the secretion of AMPs from *C. reinhardtii* includes many natural advantages, such as glycosylation for proteins to stabilize their structure and biological activities [45,46], the utilization of algae as a byproduct [47], and the large-scale cultivation in photoreactors for industrial production [48,49], there are few reports about the secretion of AMPs by *C. reinhardtii*. In previous studies, the signal peptides ARS1 and CAH1 were confirmed to have a secretory expression function and could be used in various areas such as in the delivery of targeted drugs [50] and the secretion of human intrinsic factors [51], xylanase 1 [27], and luciferase [31]. In this study, “ARS1-ALF*Pm*3” and “CAH1-ALF*Pm*3” expression cassettes were designed. Based on genomic PCR, RT-PCR, and immunoblot analyses, transgenic algal cells that could express and secrete ALF*Pm*3 peptide in *C. reinhardtii* were obtained. It was noted that, according to the immunoblot assay using the medium supernatant, only one out of three transformants could secrete the protein into the medium, which might be because of the random integration of foreign DNA into the nucleus of *C. reinhardtii* [52]. In previous studies, it was observed that foreign DNA could be cleaved during transformation [53,54]. The resulting fragments could induce complex situations, including deletions and inversions of genomic DNA flanking the foreign DNA [54,55,56] and the integrated concatemers of identical molecules in transformants with multiple copies of foreign DNA [54,57]. The above events might cause gene silencing or transcript instability [57,58,59], thereby affecting the expression and secretion of the target protein [60]. Compared with ALF*Pm*3 secreted by *P. pastoris* [42], a-ALF*Pm*3 and c-ALF*Pm*3 secreted by *C. reinhardtii* in this study exhibited a strong inhibitory ability against *V. harveyi*, *V. alginolyticus*, *V. anguillarum*, and *V. parahaemolyticus* within 24 h, and the inhibition rate was much higher than that of ampicillin (2 mg/mL). 

Differences in signal peptides affect the secretion yield and activity of recombinant proteins distinctly by influencing the folding state of the peptide chain [61,62], translocation efficiency [63], and protein stability [64,65]. In the study of signal peptides mediating the secretion and expression of NHases in *B. subtilis*, it was found that the values of the highest secretion were nearly 4.32 those of the lowest secretion [66]. The human–mouse chimeric CMV-IgM was successfully expressed and secreted in CHO cells by five signal peptides, and its secretory efficiency was more than 6.72-fold different [67]. According to the previous research, when fused with different signal peptides, the secretion yield of mCherry fluorescent proteins in *C. reinhardtii* was up to 10-fold different [29]. In this study, comparing a-ALF*Pm*3 with c-ALF*Pm*3, the secretion yield of c-ALF*Pm*3 was 2.06 times that of a-ALF*Pm*3, and the inhibition rate of c-ALF*Pm*3 was also much higher than that of a-ALF*Pm*3, indicating that both the signal peptides could secrete the ALF*Pm*3 peptide out of *C. reinhardtii* efficiently, and the CAH1 signal peptide performed better than the ARS1 signal peptide. In particular, ALF*Pm*3, derived from T-JcA1, had an inhibitory rate of over 98% against four pathogenic bacteria within 24 h, indicating that the strategy of “CAH1-ALFPm3” constructed in this study had a high potential value of AMPs.

This work provides a new idea to produce ALF*Pm*3 with high antibacterial activity. In the future, further improvements in the secretion efficiency of T-JaA and T-JcA will be conducted, and the application value of ALF*Pm*3 in the aquaculture industry will be further explored.

## 4. Materials and Methods

### 4.1. Bacterial Strains, Algal Strain, and Culture Conditions

The *C. reinhardtii* cell-wall-deficient strain JUV was purchased from the Chlamydomonas Resource Center (Duke University, Durham, NC, USA). It is suitable for cultivation in triethyl phosphate (TAP) liquid medium or TAP agar solid medium with 100 µg/mL ampicillin (Biosharp, Anhui, China) at a temperature of 22–25 °C and a light intensity of 90 μE·m^−2^·s^−1^. Transgenic algae were screened and cultivated on TAP liquid or agar solid media containing 100 µg/mL ampicillin and 10 µg/mL zeocin.

Bacterial strains of *V. harveyi*, *V. alginolyticus*, *V. anguillarum*, and *V. parahaemolyticus* were kindly provided by Professor Huang Jianhua from the South China Sea Fisheries Research Institute of the Chinese Academy of Fishery Sciences. Bacteria were cultured on LB medium under 37 °C at 200 rpm.

### 4.2. Plasmid Construction and Genetic Transformation

**Figure 5:**
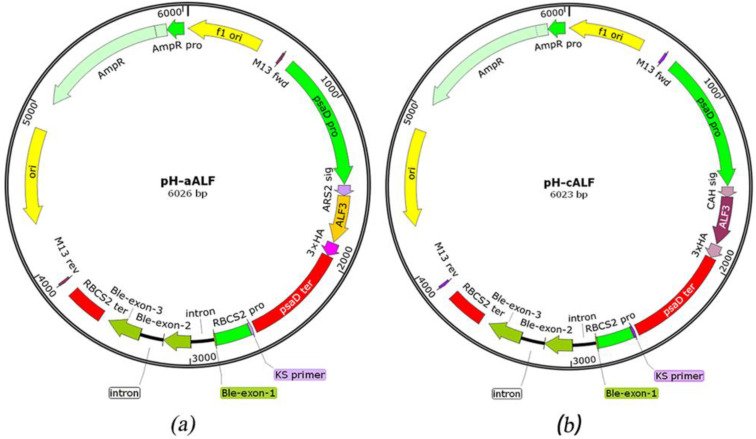
The sketch map of constructed plasmid used in this study. (**a**) The plasmid of pH-aALF. (**b**) The plasmid of pH-cALF.

ARS1 signal peptide [27] and CAH1 signal peptide [31] were selected to construct the fusion protein with ALF*Pm*3 from *P. monodon*. The fusion fragments were named ARS1-ALF*Pm*3 and CAH1-ALF*Pm*3, respectively. In order to improve the transformation efficiency and enhance the heterologous expression of *C. reinhardtii*, the coding sequence of ALF*Pm*3 (Genbank number: JQ256520) was optimized based on the codon bias of the genome of *C. reinhardtii*. Subsequently, fusion fragments tailed with 3 × HA tag were inserted into the expression vector pESVH to obtain the plasmid pH-aALF (Figure 5a) and the plasmid pH-cALF (Figure 5b). The “ARS1-ALF*Pm*3-3 × HA tag” and “CAH1-ALF*Pm*3-3 × HA tag” expression cassettes were driven by the pasD promoter and the pasD terminator. The RBCS2 promoter and the RBCS2 terminator were used to drive the expression of *sh-ble* given the transformants with zeocin resistance. The transformation of *C. reinhardtii* JUV was performed using the glass bead method according to the literature [68]. Transformed *C. reinhardtii* cells were selected on a TAP agar plate containing 100 µg/mL ampicillin and 10 µg/mL zeocin at 22 °C, with continuous light intensity 90 μE·m^−2^·s^−1^ for 2–3 weeks until green colonies appeared. The number of colonies was recorded and used to calculate the transformation frequency.

### 4.3. Genomic PCR and RT-PCR Analysis

Genomic PCR and RT-PCR were performed to identify the positive transformants [69] using 2 × M5 HiPer plus Taq HiFi PCR mix (Mei5bio, Beijing, China) as recommended. For genomic PCR, algal cells were collected from 2 mL cell culture by centrifugation at 5000 rpm for 10 min and then subjected to genomic DNA extraction using the Ultra DNA Isolation kit (Beibei Biotechnology Co., Ltd., Zhengzhou, China). Then, 2 microliters of extracted DNA was used in the 20 μL PCR reaction system with the primer pair psaD-P/psaD-T (Table 1). The PCR program was 95 °C for 3 min, 35 cycles of 94 °C for 25 s, 58 °C for 25 s, 72 °C for 40 s, and finally extended at 72 °C for 5 min. For RT-PCR, the total RNA was extracted with an RNA fast 200 kit (Fastagen, Shanghai, China), and the first stranded cDNA was synthesized using a Hifair^®^ III 1st Strand cDNA Synthesis SuperMix for qRCR (gDNA digest plus) (Yeasen, Shanghai, China), as recommended by the instructions. The RT-PCR reaction system was in a 20 μL column containing 4 μL cDNA as the template. The amplification primer pair for T-JaA was Fa/ALF3R1, and that for T-JcA was Fc/ALF3R2. The primer pair actin-F/actin-R was selected to amplify β-actin as the internal control (Table 1). The RT-PCR program was 95 °C for 3 min, 35 cycles of 95 °C for 30 s, 58 °C for 30 s, 72 °C for 40 s, and finally extended at 72 °C for 5 min. Finally, all PCR products were analyzed via 1.5% agarose gel electrophoresis.

### 4.4. Protein Extraction and Immunoblot Analysis

To evaluate the presence of the protein ALF*Pm*3 in T-JaA and T-JcA, the total proteins in cells and the secretory protein in culture supernatant were extracted and subjected to immunoblot analysis. Algal cells were pelleted by centrifugation and total intracellular proteins were extracted based on the reference literature [9]. The extracellular protein from culture medium was extracted as follows: the culture medium was collected by centrifugation and then freeze dried. The obtained powder was finally dissolved with 1 × PBS (Biyuntian Biotechnology Co., Ltd., Shanghai, China). 

The protein was separated by 12.5% SDS-PAGE (GenScript, Piscataway, NJ, USA) and transferred to a PVDF membrane (Merck, Rahway, NJ, USA). The hybridization was performed first with Anti-HA.11 Epitope Tag Antibody (1:2000) (Biogene, Beverly Hills, CA, USA) and then with Anti-mouse IgG, HRP-linked Antibody (1:2000) (Cell Signaling Technology, Boston, MA, USA) as per the instructions. Finally, BeyoECL Moon (Biyuntian Biotechnology Co., Ltd., Shanghai, China) was used for the stain development, and Odyssey^®^ Fc (Gene Company Limited, Hong Kong, China) was used to detect chemiluminescence signals.

### 4.5. Antibacterial Assay

T-JaA6 and T-JcA1 algal cells were cultivated at 22–25 °C with continuous light intensity at 90 μE·m^−2^·s^−1^ until the cell density reached 1 × 10^6^ cells/mL. Total proteins were extracted from culture supernatant as described above and dissolved in 1 × PBS for subsequent antibacterial assay. The antibacterial assay was tested on four common aquaculture pathogenic bacteria including *V. harveyi*, *V. alginolyticus*, *V. anguillarum*, and *V. parahaemolyticus*. The tested strains were cultured with 3 mL fresh LB overnight at 37 °C, 200 rpm. Then, 30 μL overnight culture was inoculated to 3 mL of fresh LB and incubated for 1 h at 37 °C. Subsequently, the mixture was diluted 1000 times with fresh LB liquid medium for the antibacterial assay. The 50 μL protein extracts from the culture supernatant of T-JaA and T-JcA were mixed with 150 μL bacteria culture in a sterile 96-well plate. At the same time, 10 μL 2 mg/mL Ampicillin and 50 μL protein extracts from the culture supernatant of *C. reinhardtii* JUV were set as the positive and negative control, respectively. The total 200 μL mixtures were incubated at 37 °C for 24 h. During this period, the absorbance value of the mixture at 600 nm was measured every 2 h. The growth rate (∆OD_600_) of the bacteria in each treatment group was obtained by comparing the OD_600_ value at different time points with that at 0 h. The bacteriostatic rate at each time point was calculated as (%) = (ΔOD_600_ of WT − Δ OD_600_ of Experimental group)/ΔOD_600_ of WT × 100.

### 4.6. Statistical Analysis

Each experiment had three independent replicates, and the software GraphPad Prism 8 was used to perform statistical analysis and make charts. In this study, the t-test was used to analyze the statistical differences between the treatment groups. *p* < 0.05 was considered to have a significant difference. 

## 5. Conclusions

In this study, the signal peptides ARS1 and CAH1 were fused with ALF*Pm*3, respectively, and inserted into the pESVH vector to construct pH-aALF and pH-cALF plasmids, which were transformed to *C. reinhardtii* JUV using the glass bead method. The positive transformants were obtained after bleomycin resistance selection, DNA-PCR, and RT-PCR analysis. The peptide ALF*Pm*3 was detected within cells and culture supernatant, demonstrating the successful expression and secretion of ALF*Pm*3 using *C. reinhardtii*. In addition, ALF*Pm*3 extracted from the culture supernatant of T-JaA and T-JcA showed the significant inhibition of the growth of *V. harveyi*, *V. alginolyticus*, *V. anguillarum*, and *V. parahaemolyticus* within 24 h and performed much better than ampicillin (2 mg/mL), indicating the superior antibacterial activity of ALF*Pm*3. Interestingly, we found the inhibitory rate of c-ALF*Pm*3 against the tested Vibrio was 2.77 to 6.23 times as that of a-ALF*Pm*3, showing the superiority of the CAH1 signal peptide for the secretory expression of ALF*Pm*3 in *C. reinhardtii*. This study provided a new strategy for the highly efficient secretion of ALF*Pm*3 with high antibacterial activity in *C. reinhardtii*.

## Figures and Tables

**Table 1:** Sequence of primers used in this study.

Name	Sequence ( 5′–3′)	Target Gene	Expected Product (bp)
psaD-P	GGGAATTGGAGGTACGACCGAGAT	*ARS1-ALFPm3* *CAH1-ALFPm3*	679 676
psaD-T	AGCTCCGATCCCGTATCAATCAGC		
Fa	CGCGCTGGCTGTGTTCG	*ARS1-ALFPm3*	222
ALF3R1	GTCCAGCCGGGGCACCACATG		
Fc	CGCGCACCGGGGCACTCCTG	*CAH1-ALFPm3*	203
ALF3R2	ACATGCGGCCCTTGTAGTACACCT		
actin-F	ACCCCGTGCTGCTGACTG	*β-actin*	351
actin-R	ACGTTGAAGGTCTCGAACA		

## Data Availability

The original data are available from the correspondent author on request.
